# 免疫检查点通过代谢途径介导肿瘤免疫调控

**DOI:** 10.3779/j.issn.1009-3419.2025.106.08

**Published:** 2025-03-20

**Authors:** Weiguang DU, Xiyang TANG, Yulong ZHOU, Mengchao LI, Ze JIN, Jiaqi DOU, Jinbo ZHAO

**Affiliations:** ^1^710021 西安，西安医学院研究生部（杜伟光）; ^1^Graduate School of Xi'an Medical University, Xi'an 710021, China; ^2^710038 西安，空军军医大学唐都医院胸腔外科（唐希阳，周玉龙，李梦超，赵晋波）; ^2^Department of Thoracic Surgery, Tangdu Hospital, AirForce Medical University, Xi'an 710038, China; ^3^710032 西安，空军军医大学基础医学院（金泽，窦家琪）; ^3^College of Basic Medicine, Air Force Medical University, Xi'an 710032, China

**Keywords:** 免疫检查点, 肿瘤微环境, 免疫代谢, 肿瘤免疫, Immune checkpoints, Tumor microenvironment, Immune metabolism, Tumor immune

## Abstract

免疫检查点包括一系列在免疫细胞的增殖、活化以及免疫调控反应中发挥关键作用的受体-配体对。尽管目前免疫检查点抑制剂（immune checkpoint inhibitors, ICIs），如程序性死亡受体-1（programmed death protein 1, PD-1）、细胞毒性T淋巴细胞相关蛋白4（cytotoxic T-lymphocyte-associated protein 4, CTLA-4）在临床中已取得较好的疗效，但部分患者仍然存在治疗无效和免疫耐药的问题。已有大量证据表明，免疫检查点蛋白在免疫调节过程中与细胞代谢相关。一方面免疫检查点连接改变肿瘤细胞代谢重编程竞争免疫细胞所需的营养，另一方面免疫检查点通过调节免疫细胞代谢通路，如磷脂酰肌醇3激酶/蛋白激酶B/雷帕霉素靶蛋白（phosphatidylinositol 3-kinase/protein kinase B/mammalian target of rapamycin, PI3K/AKT/mTOR）影响免疫细胞的激活。基于对既往文献的回顾，本文对PD-1、CTLA-4、T细胞免疫球蛋白和ITIM结构域蛋白（T cell immunoreceptor with Ig and ITIM domains, TIGIT）、T细胞免疫球蛋白和黏蛋白结构域蛋白3（T cell immunoglobulin and mucin domain-containing protein 3, TIM-3）、分化簇47（cluster of differentiation 47, CD47）、吲哚胺2,3-双加氧酶1（indoleamine 2,3-dioxygenase 1, IDO1）如何调控细胞代谢重编程的机制进行综述，并且对靶向免疫检查点配体-受体对的“双重调控”及抑制代谢途径是否有效解决肿瘤免疫耐药的问题进行展望。

## Abstract (en)

Immune checkpoints include a series of receptor-ligand pairs that play a key role in the proliferation, activation, and immune regulatory responses of immune cells. Although immune checkpoint inhibitors (ICIs), such as programmed death protein 1 (PD-1) and cytotoxic T-lymphocyte-associated protein 4 (CTLA-4) have achieved good therapeutic effects in clinical practice, some patients still experience ineffective treatment and immune resistance. A large amount of evidence has shown that immune checkpoint proteins are related to cell metabolism during immune regulation. On the one hand, immune checkpoints connect to alter the metabolic reprogramming of tumor cells to compete for nutrients required by immune cells. On the other hand, immune checkpoints regulate the metabolic pathways of immune cells, such as phosphatidylinositol 3-kinase/protein kinase B/mammalian target of rapamycin (PI3K/AKT/mTOR) to affect the activation of immune cells. Based on a review of the literature, this article reviews the mechanisms by which PD-1, CTLA-4, T cell immunoreceptor with Ig and ITIM domains (TIGIT), T cell immunoglobulin and mucin domain-containing protein 3 (TIM-3), cluster of differentiation 47 (CD47), and indoleamine 2,3-dioxygenase 1 (IDO1) regulate cell metabolic reprogramming, and looks forward to whether targeting the ligand-receptor pairs of immune checkpoints in a "dual regulation" manner and inhibiting metabolic pathways can effectively solve the problem of tumor immune resistance.

随着对癌症的不断探索，免疫系统对肿瘤发生发展发挥着复杂的功能，一方面免疫细胞可自主识别并杀伤肿瘤细胞，另一方面肿瘤细胞通过多种机制来躲避免疫系统的监视来促进自身进展，实现免疫逃逸。在过去的几十年间，发现了许多免疫受体和配体，在调节免疫和维持免疫稳态方面提供了关键检查点。这些共刺激或共抑制蛋白可以在免疫系统中调高信号（共刺激）或调低信号（共抑制）被称为免疫检查点蛋白，在癌症免疫反应中充当守门员的分子^[1,2]^。

除了程序性死亡受体-1（programmed death protein 1, PD-1）、细胞毒性T淋巴细胞相关蛋白4（cytotoxic T-lymphocyte-associated protein 4, CTLA-4），越来越多的免疫检查点得以发现，如T细胞免疫球蛋白和ITIM结构域蛋白（T cell immunoreceptor with Ig and ITIM domains, TIGIT）、淋巴细胞活化基因3（lymphocyte activation gene 3, LAG-3）、T细胞免疫球蛋白和黏蛋白结构域蛋白3（T cell immunoglobulin and mucin domain-containing protein 3, TIM-3）等，这些免疫细胞上的共抑制分子的过表达或共刺激分子的缺失导致肿瘤细胞发生免疫逃逸，因为免疫细胞可能无法接收到激活和增殖的信号，或被抑制性检查点蛋白抑制。抑制免疫抑制检查点蛋白可以增强免疫应答，防止癌症进展，并提高患者生存率^[3]^。目前免疫检查点抑制剂（immune checkpoint inhibitors, ICIs）在临床中展现出很好的疗效，例如抗PD-1药物帕博利珠单抗和纳武利尤单抗、抗CTLA-4药物伊匹木单抗在肺癌及黑色素瘤中取得了显著的临床疗效^[4,5]^，另外部分ICIs，如TIM3抑制剂MBG-453、TIGIT抑制剂MTIG7192A、分化簇47（cluster of differentiation 47, CD47）抑制剂ALX148也处于临床试验阶段并起到一定的疗效^[6⇓-8]^，但会伴随有免疫耐药、免疫相关不良反应等问题。这些可能是免疫检查点与免疫代谢、免疫浸润之间的串联导致的。

ICIs的出现为癌症治疗带来了新希望，有效解除免疫系统的抑制机制，恢复T细胞对肿瘤杀伤功能，能为癌症患者带来更长久的生存获益。基于对既往文献的回顾，本文对PD-1、CTLA-4、TIGIT、TIM-3、CD47和吲哚胺2,3-双加氧酶1（indoleamine 2,3-dioxygenase 1, IDO1）如何调控细胞代谢重编程的机制进行了综述，并且对靶向免疫检查点配体-受体对的“双重调控”及抑制代谢途径是否有效解决肿瘤免疫耐药的问题进行展望。

## 1 免疫细胞的代谢重编程

营养竞争是肿瘤细胞发生免疫逃逸的关键代谢机制。肿瘤细胞通过“Warburg”效应，即在氧气充足的条件下，也可大量摄取葡萄糖，并将其转化为乳酸以获取能量，这一过程促进了肿瘤细胞的快速增殖和长期存活^[9]^。肿瘤细胞大量消耗营养物质导致肿瘤微环境（tumor microenvironment, TME）内的营养供应不足。这种营养剥夺现象恶化了免疫细胞的生存环境，迫使免疫细胞调整其代谢方式，包括对葡萄糖、氨基酸和脂质的利用，以适应营养受限的条件。这种免疫细胞重编程的过程中免疫细胞分化或极化为免疫抑制表型，从而使免疫细胞失去抗肿瘤功能^[9,10]^。另外免疫细胞（如淋巴细胞）也使用“Warburg效应”来满足自身的能量需求。静止的幼稚T细胞可以通过氧化磷酸化（oxidative phosphorylation, OXPHOS）的方式获取能量，会利用葡萄糖、氨基酸以及脂肪酸来满足自身的生长和运作需求。在适当的共刺激环境中识别抗原时，这些细胞则改变代谢方式以适应增加的能量需求^[3]^。

## 2 免疫检查点蛋白参与免疫细胞的代谢重编程

TME内营养成分的变化使免疫细胞进行代谢重编程，抑制免疫细胞的发育和激活，进而降低肿瘤免疫调控作用，促使肿瘤细胞的免疫逃避^[11]^。免疫检查点蛋白与免疫细胞的代谢活动密切相关。抑制免疫检查点可以通过抑制肿瘤细胞代谢促进TME中营养成分的增多，使免疫细胞通过营养竞争获取更多的养分用于自身发育及发挥抗肿瘤功能，另外免疫细胞及其效应器功能的激活依赖于导致代谢重编程的不同信号通路的激活，而这些信号通路的激活可能受免疫检查点的调控，本文除了关注PD-1、程序性死亡配体1（programmed cell death ligand 1, PD-L1）及CTLA-4在代谢途径中的作用，同样发现TIGIT、TIM-3、CD47、IDO1在代谢途径中也起到关键调节作用（图1）。

**图1:**
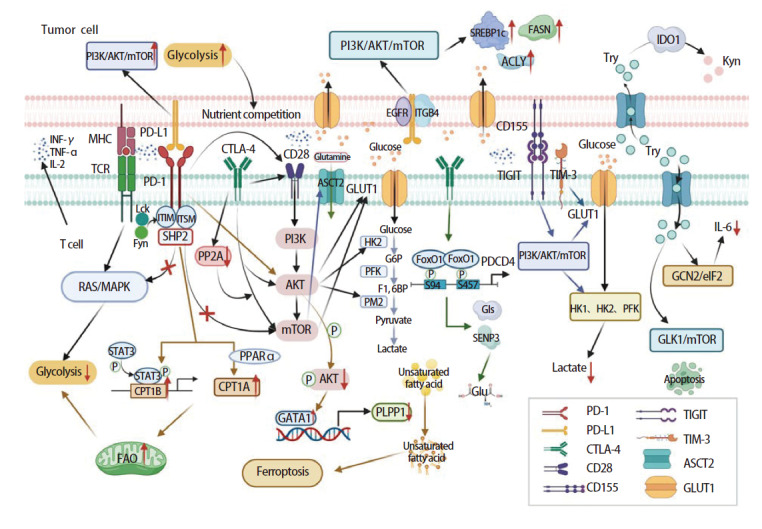
免疫检查点调控T细胞营养代谢示意图

### 2.1 PD-1/PD-L1

PD-1是一种免疫调节分子，为I型跨膜蛋白，在活化的T细胞、B细胞和自然杀伤（natural killer, NK）细胞表面表达。其配体PD-L1在肿瘤细胞、T细胞、B细胞和巨噬细胞上表达^[2]^。PD-1与PD-L1连接降低了对肿瘤细胞的免疫应答，PD-1还是T细胞在持续激活的条件下（如癌症）发生的代谢功能障碍状态（称为“T细胞耗竭”）的中心调节因子^[12]^。

PD-1/PD-L1与免疫调节过程中的糖代谢有关。既往研究^[13]^显示，PD-1与PD-L1互相作用后，Src家族激酶（Lck、Fyn）促使PD-1尾部的免疫受体酪氨酸基转换基序（immunoreceptor tyrosine-based switch motif, ITSM)和免疫受体酪氨酸基抑制基序（immunoreceptor tyrosine-based inhibitory motif, ITIM）位点酪氨酸磷酸化，招募含Src同源2结构域的蛋白酪氨酸磷酸酶2（Src homology 2 domain-containing protein tyrosine phosphatase 2, SHP2），进而下调T细胞受体（T cell receptor, TCR）信号参与的Ras/MAPK通路和CD28共刺激信号参与的磷脂酰肌醇3激酶/蛋白激酶B/雷帕霉素靶蛋白（phosphatidylinositol 3-kinase/protein kinase B/mammalian target of rapamycin, PI3K/AKT/mTOR）通路，这两条途径是T细胞糖酵解的关键调节因子，而PD-1的上调抵消了这些途径，使T细胞的糖酵解能力下降，影响其增殖与活化的能力^[12,14]^。髓系祖细胞在肿瘤早期介导的紧急骨髓生成过程中累积并产生髓源性抑制细胞（myeloid-derived suppressor cells, MDSC），这一过程与PD-1的表达有关，PD-1缺陷的髓系祖细胞响应粒细胞集落刺激因子（granulocyte-colony stimulating factor, G-CSF）增强了Erk1/2和mTORC1的激活，诱导固醇调节元件结合蛋白1（sterol regulatory element binding protein 1, SREBP1）上调，增加脂肪酸和胆固醇合成相关酶的转录，从而导致甲羟戊酸通路的糖酵解调节激活，MDSC积累减少，分化的炎症效应单核细胞输出增多，促进T_EM_细胞分化及全身抗肿瘤免疫^[15]^。PD-1是T细胞肿瘤转化过程中抑制糖酵解的关键守门员，缺乏PD-1表达的T细胞通过PI3K/AKT/mTOR/HIF1α轴诱导葡萄糖摄取和代谢的限速因子，从而加强肿瘤细胞能量的产生。此外，T细胞上PD-1的缺失会导致腺苷酸三磷酸（adenosine triphosphate, ATP）柠檬酸裂解酶（ATP citrate lyase, ACLY）的释放，触发线粒体外产生乙酰辅酶A（coenzyme A, CoA），以介导组蛋白从头乙酰化到开放染色质，并增强致癌激活蛋白1（activator protein-1, AP-1）家族转录因子的活性，加剧T细胞恶性转化及肿瘤免疫逃逸^[16]^。另外，肿瘤细胞上PD-L1的表达促进肿瘤细胞中的糖酵解和AKT/mTOR激活，同时通过葡萄糖竞争抑制T细胞中的mTOR活性，抑制T细胞对肿瘤细胞的免疫杀伤^[17]^。

PD-1/PD-L1与免疫调节过程中的脂质代谢紧密相关。PD-1与PD-L1结合后抑制T细胞糖酵解，但可以上调脂肪甘油三酯脂肪酶（adipose triacylglyceride lipase, ATGL）和肉碱棕榈酰转移酶1A（carnitine palmitoyltransferase 1A, CPT1A）的表达，促进内源性脂质的脂肪酸氧化（fatty acid oxidation, FAO），使T细胞能够利用脂肪代谢提供能量存活，减缓T细胞耗竭^[18]^。此外，PD-1连接会使得信号转导和转录激活因子3（signal transducer and activator of transcription 3, STAT3）磷酸化，促进肉碱棕榈酰转移酶1B（carnitine palmitoyltransferase 1B, CPT1B）上调。由于CPT1B是FAO的关键限速酶编码基因，进而导致脂肪酸的分解代谢加快，对糖酵解过程以及CD8^+^ T效应细胞的功能产生抑制作用^[19]^。在肺癌中CD8^+ ^T细胞上的PD-1连接抑制AKT信号通路，使转录因子GATA1发生核转位抑制磷脂磷酸酶1（phospholipid phosphatase 1, PLPP1）的转录，PLPP1表达降低导致磷脂代谢异常，使TME中的不饱和脂肪酸在进入细胞后代谢为大量的不饱和磷脂，促使CD8^+^ T细胞发生铁死亡，减少细胞因子分泌，使得对肿瘤细胞的免疫反应降低^[20]^。在肿瘤细胞表面PD-L1与表皮生长因子受体（epidermal growth factor receptor, EGFR）和整合素β4（integrin beta-4, ITGB4）形成复合物，驱动PI3K/AKT/mTOR/SREBP1c信号传导通路，进而上调脂肪酸合成酶（fatty acid synthase, FASN）和ACLY等脂肪生成酶的表达来促进肿瘤细胞脂质代谢，同时肿瘤细胞表面会分泌包含有PD-L1的外囊泡，与T细胞上PD-1连接后，激活cAMP反应元件结合蛋白（cAMP response element-binding protein, CREB）和STAT信号传导，上调胆固醇合成和磷脂调节的关键合成酶，从而促进了T 细胞的脂质代谢及T细胞衰老^[21,22]^。在上皮性卵巢癌中肿瘤相关巨噬细胞（tumor-associated macrophages, TAMs）衍生的PD-L1^+^外泌体影响转录因子氧化物酶体增殖物激活受体α（peroxisome proliferator-activated receptor α, PPARα），进而上调CD8^+^ T细胞中CPT1A的表达，促进脂肪酸氧化，增加活性氧导致细胞损伤，抑制T细胞分泌炎性细胞因子干扰素-γ（interferon gamma, INF-γ），促使肿瘤发生腹膜转移^[23]^。

PD-1在T细胞激活时影响其氨基酸代谢。PD-1与PD-L1结合后通过降低mTOR活性，减少谷氨酰胺转运体2（alanine-serine-cysteine transporter 2, ASCT2）的表达，进而抑制谷氨酰胺和支链氨基酸的转运和分解，导致T细胞抗肿瘤能力下降^[18]^。

### 2.2 CTLA-4

CTLA-4属免疫球蛋白超家族成员，在活化T细胞和Tregs表面表达，在调节T细胞活化中起着关键作用，维持免疫稳态^[2]^。通过与共刺激受体CD28竞争抗原提呈细胞上的配体B7-1和B7-2，CTLA-4传递抑制信号，限制 T 细胞的增殖和功能。

CD28可通过AKT依赖性和AKT非依赖性的方式调节T细胞中葡萄糖转运蛋白1（glucose transporter 1, GLUT1）的表达和葡萄糖摄取，进而影响T细胞的活化^[24]^。既往研究^[25]^表明CTLA4与B7蛋白结合后可跨内吞B7分子，抑制CD28信号转导通路进而剥夺刺激信号转导，同样，CTLA-4降低蛋白磷酸酶2A（protein phosphatase 2A, PP2A）的活性来抑制PI3K/AKT/mTORC1信号传导，使AKT/mTORC1活性下降，下调GLUT1、己糖激酶2（hexokinase 2, HK2）和丙酮酸激酶同工酶M2（pyruvate kinase isozyme type M2, PKM2）等糖酵解关键酶，阻止活化诱导的糖酵解，从而降低活化T细胞变成完全分化效应细胞的倾向^[13,26]^。阻断Tregs细胞上高表达的CTLA-4促使CD28共刺激信号的传出，增加了Tregs对TME中葡萄糖的可用性，使Tregs的不稳定性上升，减少IFN-γ和肿瘤坏死因子-α（tumor necrosis factor-alpha, TNF-α）的分泌，降低了Tregs对效应T细胞的抑制作用，促使效应T细胞激活而发挥抗肿瘤免疫功能^[27]^。同样有研究^[28]^发现CTLA-4的激活能够抑制c-Myc的表达，c-Myc缺陷的T细胞导致HK2、PKM2等下调，影响糖酵解活性，导致T细胞生长增殖受阻。

细胞毒性T淋巴细胞（cytotoxic T lymphocytes, CTLs）在清除肿瘤细胞中发挥关键作用，CTLA-4可以调控T细胞氨基酸代谢抑制CTLs的分化倾向^[29]^。CTLA-4消除了AP-1家族转录因子Fos相关抗原2（Fos-related antigen 2, Fra-2）的磷酸化，并导致中枢转录因子FoxO1的AKT非依赖性核重新定位，抑制PDCD4在S94和S457位点的强磷酸化，PDCD4表达下调促使谷氨酰胺酶（glutaminase, GLS）和Sentrin特异性蛋白酶3（sentrin protease 3, SENP3）合成减少，进一步使CTLs中谷氨酸的含量下降，谷氨酸可以促使野生型CD8^+^ T细胞分泌INF-γ，进而影响CTLs的分化倾向^[29]^。

### 2.3 TIGIT、CD47、TIM-3

TIGIT是蛋白超家族成员之一，TIGIT主要由T细胞和NK细胞表达。CD155为TIGIT的配体，在肿瘤细胞上表达，与TIGIT有着很高的亲和力^[2,30]^。在胃癌、结直肠癌和三阴性乳腺癌中，TIGIT/CD155通路连接通过PI3K/AKT/mTOR影响其磷酸化，进一步促使T细胞中糖酵解关键酶GLUT1、HK1、HK2和磷酸果糖激酶（phosphofructokinase, PFK）的显著降低，导致T细胞葡萄糖摄取和乳酸生成减少，导致其激活和效应功能的受损，而通过阻断TIGIT/CD155可有效逆转这一现象^[31⇓-33]^。总之，TIGIT似乎能够通过抑制糖酵解来改变T细胞功能，还需进一步探索其潜在机制。

CD47作为免疫球蛋白超家族的I型整合膜蛋白，在T细胞和肿瘤细胞上都有表达。它和先天免疫细胞的信号调节蛋白α（signal regulatory protein α, SIRPα）相互作用，发出逃避抗肿瘤免疫监视的信号^[34]^。CD47也可以通过基质细胞糖蛋白血小板反应蛋白1（thrombospondin-1, TSP1）连接到T细胞上，介导T细胞的抗肿瘤功能^[35]^。既往研究^[35]^表明，CD47与TSP1相结合降低T细胞的激活和糖酵解速率，影响T细胞抗肿瘤功能，但具体机制还尚不明确。另外，在阻断CD47/SIRPα通路治疗中激活I型干扰素（type I interferon, IFN-I）信号转导，通过ISG-15以促进肿瘤细胞OXPHOS和ATP的产生及细胞外释放，ATP细胞外释放会通过P2X7受体介导的树突状细胞激活诱导抗肿瘤T细胞反应，以揭示改变肿瘤代谢重编程加强抗肿瘤免疫^[36]^。

TIM-3属于TIM家族，最初被发现是1型辅助性T细胞上的共抑制受体，可调节I型免疫应答。TIM3在免疫细胞（如单核细胞、NK、CD4^+^ T和CD8^+^ T细胞）的表面高表达 ，并在TME中发挥免疫调节作用^[34,37]^。TIM-3的高表达提示肿瘤预后不良可能与调节T细胞代谢重编程有关。既往发现TIM-3促进Jurkat细胞GLUT1的表达、葡萄糖摄取和乳酸释放，从而影响T细胞对肿瘤的免疫效能，这可能与PI3K/AKT通路有关^[38]^。同样TIM-3还参与巨噬细胞的糖酵解，在RAW264.7小鼠巨噬细胞中TIM-3通过抑制STAT1信号促使HK2的表达下调，导致巨噬细胞的糖酵解受损，进而影响巨噬细胞表型及炎症因子的释放^[39]^。

### 2.4 IDO1

IDO1作为新型免疫检查点蛋白，主要在肿瘤细胞（肺癌、黑色素瘤、肾细胞癌等）、抗原提呈细胞和MDSC等细胞上表达，IDO1本质上是一种限速酶，其介导的色氨酸（tryptophan, Try）代谢途径的改变会对免疫细胞的代谢重编程产生显著影响^[34,40]^。Try的耗竭将导致免疫细胞内代谢途径的重新配置，进而对T细胞的效应功能产生抑制作用。

IDO1在肿瘤细胞中通过促进Try的分解，大量消耗Try，使T细胞利用降低，导致一般控制非阻遏蛋白2（general control nonderepressible 2, GCN2）的激活，GCN2是应激反应激酶，可灭活翻译起始因子2（eukaryotic initiation factor 2, eIF2）使促炎细胞因子白介素-6（interleukin-6, IL-6）在内的多个基因表达失调，另Try的缺失还导致GLK1/mTOR信号通路的下调，诱导T细胞自噬，使效应T细胞无反应^[41,42]^。Campesato等^[43]^发现高表达IDO1的黑色素瘤肿瘤中，IDO1能够诱导Try代谢产物犬尿氨酸（kynurenine, Kyn）的生成，Kyn作为内源性配体，具备激活芳烃受体（aryl hydrocarbon receptor, AHR）信号传导的能力。Tregs通过AHR信号的激活可以调控M2型肿瘤相关巨噬细胞（M2-type tumor-associated macrophages, M2-TAMs）的功能，M2-TAMs能够分泌多种免疫抑制因子，如白细胞介素-10（interleukin-10, IL-10）、转化生长因子β（transforming growth factor-beta, TGF-β）等，对CD8^+^ T细胞的免疫反应产生抑制效果。总体来说 IDO-Kyn-AHR参与Tregs-巨噬细胞抑制轴来抑制TME中的T细胞免疫调节^[43]^。

## 3 免疫检查点之间的调控机制

如上所述，不同的免疫检查点通过调控肿瘤细胞和免疫细胞的代谢重编程来影响免疫功能，然而它们之间存在复杂的相互作用和调控关系。不同免疫检查点对细胞代谢途径的调控存在共同点和差异，例如PD-1和CTLA-4的高表达与T细胞的耗竭密切相关，在这种状态下T细胞的代谢功能受到抑制，无法有效地发挥其抗肿瘤作用；PD-1、CTLA-4、TIGIT、TIM-3都主要是以PI3K/AKT/mTOR信号通路作为主要代谢通路，抑制T细胞代谢活性，进而影响T细胞增殖与激活；另外免疫检查点的作用阶段不同，如CTLA-4通过早期抑制CD28的刺激信号来抑制T细胞的初始激活，而PD-1则是在晚期通过抑制TCR信号通路来抑制T细胞功能性激活，形成了T细胞整体的代谢抑制互补^[44]^；不同免疫检查点对免疫细胞的抑制强度也是有所不同，与CTLA-4相比，PD-1对Tregs的免疫抑制则相对较小。免疫检查点间还可能存在免疫级联调控，PD-L1调控肿瘤细胞的糖酵解进而增加TME中乳酸含量，而乳酸则会促进USP39介导的RNA剪接，以Foxp3依赖性方式促进CTLA-4表达，进一步加剧免疫抑制功能^[45]^。总之不同免疫检查点在肿瘤免疫微环境中的复杂调控抑制了免疫细胞的抗肿瘤功能，进一步促使免疫逃避。

## 4 结语和展望

随着免疫检查点被发现，它们展现出了成为免疫治疗靶点的巨大潜力，并在肿瘤免疫调控中扮演着至关重要的角色。然而，对免疫检查点的调控机制仍不完全清楚。本文讨论了PD-L1、PD-1、CTLA-4、TIGIT、IDO1、TIM-3、CD47等免疫检查点蛋白如何通过直接或间接的方式改变免疫细胞的代谢重编程。免疫细胞参与肿瘤的免疫调控离不开免疫检查点所介导的细胞代谢过程，免疫检查点通过抑制免疫细胞的糖代谢、脂质代谢和氨基酸代谢，限制了免疫细胞的激活、分化、增殖以及效应功能。免疫检查点还促进肿瘤细胞的糖酵解，如PD-L1的高表达激活肿瘤细胞PI3K/AKT/mTOR通路，增加葡萄糖摄取和脂质代谢，并在存在营养竞争的情况下，免疫细胞进行代谢重编程，影响免疫功能，说明ICIs对免疫细胞及肿瘤细胞的“双重靶向”（如共阻断PD-L1/PD-1）会进一步提高免疫治疗效果。

此外，免疫细胞代谢重编程推动免疫耐药和T细胞的代谢改变，促使糖酵解转变成氧化磷酸化，减少能量利用进而损害T细胞效应功能，导致对肿瘤细胞的杀伤作用减弱，肿瘤细胞发生免疫耐药；髓系祖细胞在癌症早期驱动产生并积累MDSC，通过代谢重编程抑制免疫细胞功能，推动肿瘤免疫耐药；另外TME能量代谢导致巨噬细胞向M2型极化，分泌血管内皮生长因子促进肿瘤的增殖，同时免疫抑制细胞因子IL-10、TGF-β等抑制CD8^+^ T细胞抗肿瘤功能。改变肿瘤细胞代谢重编程可改善TME内的葡萄糖可用性，也可以保持抗肿瘤T细胞免疫并促进对ICIs的反应，如富马酸二甲酯可以抑制肿瘤细胞的有氧糖酵解，促使肿瘤细胞与T细胞间营养竞争正常化，增强肿瘤浸润性CD8^+^ T细胞的抗肿瘤免疫，从而优化ICIs治疗的疗效^[46]^。目前靶向代谢途径联合ICIs的多项试验正在进行，谷氨酰胺酶抑制剂（JHU083）联合PD-1抑制剂、聚乙二醇化精氨酸脱亚胺酶（arginine deiminase PEGylated, ADI-PEG20）（可将精氨酸分解为瓜氨酸）联合抗PD-L1抗体等展现出一定的抗肿瘤疗效^[47,48]^。因此通过靶向代谢途径有效解决单一应用ICIs所产生的耐药性问题将会是未来的重点关注方向。

免疫检查点与细胞代谢通路之间的联系错综复杂，仍有许多问题值得去探索。需要进一步探究免疫检查点在细胞内的信号传导通路与代谢调控的精确分子机制，随着对免疫检查点与代谢重编程认识的加深，将会大大提高免疫疗法的有效性。
